# Measuring the Nursing Work Environment during Public Health Emergencies: Scale Adaptation and Validation

**DOI:** 10.1155/2024/9910079

**Published:** 2024-03-05

**Authors:** Xiyi Wang, Jing Shao, Jiaqi Lian, Aozhou Weng, Jianhong Chang, Mengting Ji, Caifeng Wang, Qiong Fang, Zhihong Ye, Yun Hu

**Affiliations:** ^1^School of Nursing, Shanghai Jiao Tong University, Shanghai 200025, China; ^2^Institute of Nursing Research and Department of Nursing of Fourth Affiliated Hospital, Zhejiang University School of Medicine, Hangzhou 310058, China; ^3^SAS Institute, Inc, Cary, NC 27513, USA; ^4^Renji Hospital, School of Medicine, Shanghai Jiao Tong University, Shanghai 200127, China; ^5^Sir Run Run Shaw Hospital, Zhejiang University School of Medicine, Hangzhou 310016, China

## Abstract

**Aim:**

To develop a scale for measuring nurse's perceived work environment during the public health emergencies (PHEs) and assess its reliability and validity.

**Background:**

Although there is extensive research on instruments for measuring nursing work environments in regular healthcare settings, there is a lack of specific scales tailored to address the unique work conditions experienced by nurses during PHEs.

**Design:**

This study employed a cross-sectional design for psychometric evaluation and adhered to the Strengthening the Reporting of Observational Studies in Epidemiology (STROBE) statement.

**Methods:**

A self-report scale, the Chinese Nursing Work Environment Scale for Public Health Emergencies (C-NWE-PHE), was developed, integrating situational characteristics. Data on demographics, adapted scale scores, and subjective evaluations of nursing management performance were collected from 1156 nurses through online surveys conducted between January 2023 and March 2023. Confirmatory factor analysis, Pearson correlations, and Cronbach's alpha analyses were conducted to evaluate the psychometric properties of the scale.

**Results:**

The adapted C-NWE-PHE scale comprised 28 items organized into five subscales: Workforce and Deployment Support, Leadership and Emergency Management, Autonomy and Empowerment, Teamwork and Collaboration, and Logistics and Humanistic Care. Structural equation modelling showed satisfactory factor loadings for each subscale and a good model fit, confirming construct validity. The content validity and reliability of the total scale were confirmed.

**Conclusion:**

This study provides empirical evidence for understanding and assessing the nursing work environment during PHEs with a psychometrically sound scale. *Implications for Nursing Management*. The C-NWE-PHE scale, along with its five identified constructs, provides a nuanced comprehension of working conditions amid PHEs. Implementing this scale could foster specific enhancements, support nurse retention efforts, and enhance the effectiveness of responses during challenging emergency situations.

## 1. Introduction

The COVID-19 pandemic has sparked widespread discussions regarding the burnout and psychological well-being of nurses [1–3]. These issues have had a significant impact on nurses' commitment to the nursing profession and their contributions to fighting against public health emergencies (PHEs) [1, 4]. Nurses worldwide have reported experiencing a poor work environment during the pandemic, resulting in negative work outcomes, low job satisfaction, and increased turnover intentions [5, 6]. Numerous studies have provided compelling evidence that establishing a healthy work environment is an effective strategy for mitigating job burnout, retaining nurses, and promoting sustainable development [7–9]. Given the urgency of the matter, it is vital to explore the concept, core components, and assessment tools to guide investments in establishing supportive environments at the organizational level [5, 7, 10–13]. These initiatives should be specifically tailored to crisis management contexts and encompass generalizable managerial strategies to enhance nurses' contributions to respond to future PHEs.

Public health emergencies (PHEs) are characterized by their causes and events that pose a threat to overwhelm routine capabilities due to their scale, timing, or unpredictability [14]. The recent COVID-19 pandemic serves as a prime example of a PHE that requires international attention and emergency response [10, 12]. PHEs have underscored the importance of specific working conditions, policies, health regulations, and operational practices in effectively addressing the challenges [15]. Measures taken by various countries, such as establishing designated hospitals, mobile cabin hospitals, and temporary treatment centres, have played pivotal roles in strengthening healthcare system capacity and capabilities during the COVID-19 pandemic [15–17]. Alongside expanding functional capabilities, there have been suggestions for enabling multisectoral organizational strategies and mobilizing human resources to enforce health security capacities worldwide [15, 18]. However, these measures have also introduced additional complexities for organizations, particularly in terms of ensuring nurse engagement, supporting their well-being, and fostering sustained commitments. To prepare healthcare systems for PHEs, it is important to optimize the work environment, improve the work performance, and enhance the well-being of nurses.

The nursing work environment is a multidimensional concept that significantly influences nursing practice and patient outcomes [19]. Researchers have labelled its concept domains differently, such as work values, nurse satisfaction, perceived productivity, organizational traits, autonomy, control, collaborative relationships, patient-centred care, and organizational support, etc. [20–22]. Nurses' perceptions of their working environments during the COVID-19 pandemic have been extensively explored through qualitative interviews, revealing significant challenges such as increased workload, safety concerns, emotional stress and burnout, inadequate resources, and professional and personal sacrifices [3, 23–25]. Nurses have made remarkable displays of resilience and adaptive capacity, with factors like camaraderie, recognition, and appreciation playing a vital role in mitigating the impact on their well-being [26, 27]. To enhance working conditions and prioritize initiatives, practical actions should be taken, such as ensuring staff adequacy, competence, empowerment, and equitable distribution [27–30]. Additionally, the six Pathways to Excellence Standards, which include shared decision-making, leadership, safety, quality, well-being, and professional development, were developed and implemented [27]. However, there is still a need for a comprehensive understanding of how emergency response and managerial strategies affect the practice environment and nurse outcomes during PHEs [27, 31, 32]. Effective measures that incorporate accountability-oriented evaluations can enable external comparisons and internal efforts for improvement [14]. To gain deeper insights into the work environment during PHEs, quantitative approaches are necessary, providing a solid foundation for assessments and understanding their impact on nurse performance, thereby informing strategies to improve nursing practice and outcomes.

Previous literature reviews on measuring work environments have highlighted that existing questionnaires primarily focus on general hospital settings and evaluate physical environments within healthcare facilities [20, 33]. However, emerging evidence in the COVID-19 era underscores significant differences in nurses' needs and essential support resources, encompassing logistics, emotional well-being, patient care, teamwork, and protective trainings, all crucial elements contributing to a healthy work environment [4, 34, 35]. Moreover, recent studies have demonstrated a noteworthy link between nurses' perceptions of work conditions and job outcomes, including their perceived workload, staffing adequacy, leadership, and overall organizational performance [35–37]. Initial investigations into suitable staffing solutions and their impact on the work environment, utilizing the practice environment index in acute care settings, revealed potential predictive relationships between these variables [4, 38]. As of now, there remains a lack of a specific scale for measuring and assessing work environments in emergency situations related to PHEs. To address this gap, it is practical to adapt a self-reported assessment tool based on previous evidence in measuring nurses' work environment in normal healthcare settings, thereby enhancing domain specificity and addressing situational phenomena [39, 40]. Through modifications in item wording, the scale can be refined to align with the situational context, and further validity evidence can be gathered, typically through confirmatory factor analysis [39]. This strategy allows for the adaptation of an established assessment tool, providing deeper insights into the dynamics of the work environment, particularly within the context of PHEs.

In an effort to comprehensively assess the nursing work environment and provide insights on a global scale, a meticulously developed Chinese Nursing Work Environment (C-NWE) scale was selected for adaptation. Originating in 2010, this scale was constructed based on the principles of the person-environment fit theory and contextual factors. It amalgamated crucial elements from widely used assessments, including the professional practice environment, nursing work index-revised, and practice environment index. After evaluating empirical data within the Chinese context, researchers noticed that the job-demands resources theory, centred on external influences, didn't fully capture nurses' work environment perceptions. Consequently, the C-NWE underwent refinement, integrating the theory of harmony management to enhance the understanding of individual resources, person-environment interactions, and value alignment within the scale. Initially, a 47-item scale was developed [22], aligning with global efforts exploring nursing work environment traits while integrating the influence of nurses' individual values and empowerments on their work environment [41, 42]. For greater usability, the C-NWE was further condensed into a 26-item scale using rigorous statistical methodologies and employed in a national survey covering eight economic regions [43]. This extensive survey gathered data from 19,000 nurses across 31 provinces in mainland China [9, 43], demonstrating robust psychometric properties and considerable efficiency. The 26-item version retained elements such as professional development, clinical autonomy, salary and welfare, recognition of value, while further refining aspects related to staffing adequacy, support and care, and nurse-physician relationships, thereby improving the reflective nature of person-environment interactions. These foundational constructs provide a solid theoretical framework for the current study, guiding the analysis of data pertaining to nurses' perceived work environment during the COVID-19 pandemic.

This study seeks to address the complexities of the nursing work environment concept, especially within the context of PHEs and organizational strategies geared towards effective responses to such crises. The primary objective is to construct a dependable scale for assessing nurses' perceptions of their work environment during PHEs. Validating this scale through empirical survey data will establish a robust framework for understanding the underlying theoretical concepts. This comprehension could facilitate targeted improvements in the nursing work environment, ultimately bolstering nurse retention rates and promoting well-prepared and efficient responses to future PHEs on a global scale.

## 2. Materials and Methods

### 2.1. Design

Following Heggestad's practical guideline [39], we implemented a systematic approach to adapt a measurement scale within the situational context of the items, known as scale adaptation. The initial phase aimed to generate the scale items and accurately measure the underlying construct in the target situation. Subsequently, in the second phase, we focused on reporting supporting validity evidence specifically among nurses who had experienced an appropriate level of crisis exposure. The study findings were reported in accordance with the Strengthening the Reporting of Observational Studies in Epidemiology (STROBE) statement [44].

### 2.2. Scale Adaptation

#### 2.2.1. Item Generation

The C-NWE underwent a comprehensive redesign to cater specifically to the unique challenges posed by PHEs, resulting in the creation of the C-NWE-PHE (Chinese Nursing Work Environment Scale for Public Health Emergencies). The refinement was necessitated by the need to distinguish between a typical hospital nursing work environment and the specific demands of organizational traits for responding to an event of PHE. To thoroughly understand the situational context, an integrative literature review on nurses' experiences during the COVID-19 pandemic was conducted (unpublished), incorporating expert views and identifying the core competencies of nursing professionals in public health emergencies [45]. Specific items were generated from qualitative data obtained through semistructured interviews, enabling a secondary analysis of 17 nurse managers' experiences with crisis exposure to identify scale constructs. The goal of this process was to adapt the content of the C-NWE scale to accurately reflect emergency-specific traits and working conditions [39]. The generated 28 items were then theoretically categorized into five factors using thematic induction. The research team sought the expertise of nine researchers knowledgeable in nurses' response to PHEs and seven experts to thoroughly review and enhance the alignment between the research instrument and the contextual factors. As a result, a 28-item C-NWE-PHE scale was used for validation research. The scale response options and scoring rules in the current study remained consistent with the 26-item C-NWE scale, ranging from strongly disagree to strongly agree.

#### 2.2.2. Content Validity Verification

A panel of seven experts, consisting of five nursing professors (senior clinical scientists) and two clinical nursing deputies, was invited to evaluate the content validity of the C-NWE-PHE scale. The Delphi survey included the original 26-item C-NWE scale and an overview of its subscales, the 28-item C-NWE-PHE, and an assessment form for content equivalence. Each expert was asked to rate the relevance of each item using a scale ranging from 1 (not relevant) to 5 (very relevant and succinct). Additionally, the experts were requested to provide demographic information, such as their education level, working experience, research areas, and familiarity with the scale's topic, via e-mail. The response rate was 100%, with all experts returning the evaluation surveys. To ensure the reliability of the assessment, three coefficients were calculated: the expert familiarity coefficient (Cs) and expert judgment coefficient (Ca), which yielded values of 0.91 and 0.94, respectively. Moreover, the expert authority coefficient (Cr) was determined to be 0.93, exceeding the threshold of 0.7, indicating an acceptable level of agreement among the experts [46]. Based on the experts' feedback and assessments, the research team conducted a rigorous comparison and discussion of the results to achieve consensus and identify any necessary revisions. Subsequently, an initial version of the C-NWE-PHE scale was developed for a preliminary survey among nurses.

#### 2.2.3. Preliminary Survey

Following the cognitive validity assessment approach [40], a trained investigator invited and recruited 10 nurses who were actively serving on the frontline during the COVID-19 pandemic. These nurses were requested to evaluate the scale from a cognitive standpoint, offering detailed feedback on the comprehensibility of the scale items, relevance to their perceived work environment, and the convenience of the distribution platform employed for data collection. It took approximately 14 minutes to complete the survey.

### 2.3. Scale Validation

The validation process commenced with workshop discussions and was carried out in a scale-specific manner. Adhering to practice recommendations [39], we ensured the verification of the scale by employing a fixed set of items, a predetermined response scale, and carefully crafted instructions. We sought responses from nurses with first-hand working experience to ensure the relevance and validity of the scale. The implementation protocol for survey distribution underwent refinement through three rounds of workshops, each involving 10 members. This process resulted in a rigorous validation approach that aligns with the research objectives.

#### 2.3.1. Participants

Using purposive sampling approach, a skilled investigator facilitated the recruitment process and identified eligible nurses who met the following criteria: (1) registered nurses employed full-time in Shanghai tertiary hospitals, (2) possessing at least one year of work experience in current hospitals, (3) having prior experience with and exposure to public health emergencies such as the COVID-19 pandemic, earthquakes, Ebola, SARS, etc., and (4) expressing willingness to participate in the survey.

#### 2.3.2. Instruments

The C-NWE-PHE scale, along with a demographic questionnaire and four subjective questions, was employed to assess the work environment and performance of nursing management from the perspective of nurses. The demographic questionnaire collected information on age, religion, education level, job position, job tenure, workplace, and frontline experiences of eligible nurses. The 28-item C-NWE-PHE scale was presented in a digital format, requiring respondents to rate workplace characteristics on a 6-point scale ranging from 1 (strongly disagree) to 6 (strongly agree), consisting with the rating approach of the original 26-item C-NWE scale.

#### 2.3.3. Data Collection

Between January 2023 and March 2023, a user-friendly digital platform called Chao Xing was utilized for data collection. Chao Xing operates as a mobile learning system, comprising platform and application terminals. It includes features such as a community announcement board and a survey or evaluation mode, accessible through individual user accounts and passwords. This period followed the strict lockdown in Shanghai city experienced by nurses from April 2022 to June 2022, as well as subsequent policy changes. The data collection process was facilitated by a collaborative effort involving three investigators and one data engineer who designed a logically structured digital survey. Each user was permitted to complete only one survey, identified through IP and account authentication. The survey, including a digital survey link, inclusion criteria, and participant consent, was distributed to 12 affiliated hospitals nurses but anonymously to their nursing managers to avoid report bias. To ensure the integrity of the study, researchers maintained a blind approach to the data collection process.

### 2.4. Data Analysis

Data management was conducted using Excel, and analysis was performed using SPSS 28.0 and SAS Studio software. Two-tailed tests were conducted with a significance level set at *P* < 0.05. Descriptive analysis was utilized to report the mean and standard deviation (SD) for continuous variables and percentage frequency for categorical variables. Scale validation was conducted following the COSMIN (Consensus-based Standards for the selection of health status Measurement Instruments) checklist [47]. Content validity, including face validity, was evaluated based on expert ratings using the item-level content validity index (I-CVI) and the average scale-level content validity index (S-CVI/Ave). Construct validity was assessed through confirmatory factor analysis (CFA) using structural equation modelling (SEM). Criterion validity was examined by calculating Pearson's correlation coefficients to assess the relationship between C-NWE-PHE scores and quantitative data on the overall assessments of nursing management performance. Reliability analysis included testing the homogeneity of items using Cronbach's alpha, and test-retest reliability was assessed using the intraclass correlation coefficient (ICC) with a two-way mixed-effects model and a consistency definition [48].

### 2.5. Ethical Considerations

This study was performed in accordance with the Declaration of Helsinki. Ethical approval was obtained from the ethics committee of a university-affiliated hospital (Approval No. RA-2022-397), and the investigation protocol involving 12 affiliated hospitals was approved by the ethics committee of a university (Approval No. SJUPN-HY-202304-3-KS1). All participants gave written informed consent through a digital platform before their inclusion in the study.

## 3. Results

### 3.1. Characteristics of the Study Participants

Out of the 3000 nurses invited from 12 tertiary hospitals, a total of 1156 nurses participated and 1059 nurses (91.61%) provided valid responses for the data analysis. As shown in Table 1, the average working experience of the participants was 9.2 years. Most of the participants were female (86.8%) and held primary job positions (82.8%). Most nurses were required to work at least 5 hours per day (60.0%) and fewer than 5 days per week (75.2%). The two most common workplaces were designated hospitals (32.6%) and Fangcang shelters (29.7%).

### 3.2. Description of the C-NWE-PHE

**Figure 1:**
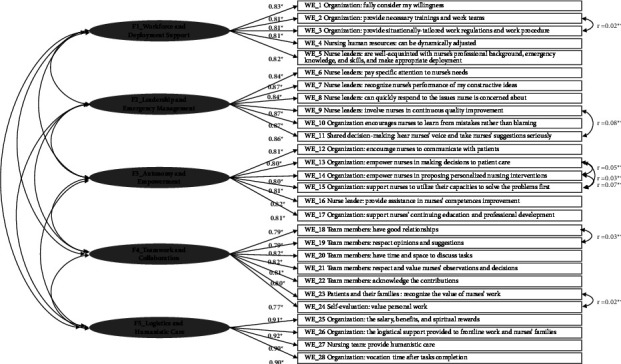
Construct of the Chinese nursing work environment scale for public health emergencies. ^∗^denotes the factor loading of each item within the corresponding theoretical factor; ^∗∗^denotes the covariance parameters identified and added to the model.

Figure 1 illustrates the internal structure of the C-NWE-PHE scale, comprising a five-factor solution, which underwent validation by the expert panel and tested through CFA. Drawing from the qualitative interview data and its conceptual interpretation, the key component of each item was presented. Each factor consisted of a varying number of items, ranging from four to six.

The five identified subscales were named in alignment with a comprehensive understanding of the nursing work environment as follows: Workforce and Deployment Support (*F*1), Leadership and Emergency Management (*F*2), Autonomy and Empowerment (*F*3), Teamwork and Collaboration (*F*4), and Logistics and Humanistic Care (*F*5). Applying the SEM method, the factor loadings of items to each subscale were found to be 0.81–0.83 for *F*1, 0.84–0.87 for *F*2, 0.80–0.82 for *F*3, 0.77–0.82 for *F*4, and 0.90–0.92 for *F*5. All subscale scores demonstrated significant loadings on the first higher-order factor (loadings > 0.49), indicating that the C-NWE-PHE scale effectively measures the various constructs of the nursing work environment during a PHE.

### 3.3. Content Validity

In comparison to the 26-item C-NWE scale, two additional items were added to the 28-item C-NWE-PHE scale: “The organization and the nursing team can fully consider my willingness to participate in emergency rescue work” and “Nursing team can provide situationally tailored work regulations and work procedures.” The C-NWE-PHE scale was validated by a 7-expert panel, showing a good I-CVI range of 0.85 to 1.00 and an overall S-CVI/Ave of 0.93. Notably, 13 items with an I-CVI of 0.85 exceeded the suggested standard of 0.83, requiring only minor revisions based on the experts' detailed suggestions. For instance, the term “doctors” in all items of the original scale was changed to “team members,” and “salary” was expanded to include salary, benefits, and spiritual rewards in the PHE context.

### 3.4. Construct Validity

The model validation was based on a hypothesized five-factor solution. Initially, a model with no covariance between any pair of items was tested. Using Lagrange Multiplier (LM) statistics and residual analysis, specific covariance parameters were identified and added to the model, resulting in an enhanced fit. The identified item pairs, along with their respective correlations and significance levels, were as follows: items 2 and 3 (*r* = 0.02, *P* < 0.001), items 9 and 11 (*r* = 0.08, *P* < 0.001), items 13 and 14 (*r* = 0.05, *P* < 0.001), items 13 and 15 (*r* = 0.03, *P* < 0.001), items 14 and 15 (*r* = 0.07, *P* < 0.001), items 18 and 19 (*r* = 0.03, *P* < 0.001), and items 23 and 24 (*r* = 0.02, *P* < 0.001).

Figure 1 displays the fit indices of the a priori theorized factor model, yielding the following results: *χ*^2^ (333) = 1822.480, *P* < 0.001, with a *χ*^2^/df ratio of 5.47. The comparative fit index (CFI) was 0.97, the Tucker–Lewis index (TLI) was 0.97, the root mean square error of approximation (RMSEA) was 0.07, the standardized root mean square residual (SRMR) was 0.02, the goodness-of-fit index (GFI) was 0.97, and the adjusted goodness-of-fit index (AGFI) was 0.86. These findings indicate that the model demonstrated a reasonably good fit to the data.

### 3.5. Reliability

Table 2 illustrates the standardized Cronbach's alpha coefficients for the total score, indicating a high internal consistency, with a coefficient of 0.99 based on 28 items. Cronbach's alpha coefficient for the subscales ranged from 0.96 to 0.98. Additionally, the test-retest reliability, assessed among 22 nurses over a two-week period, yielded a coefficient of 0.97.

## 4. Discussion

This study presents an exploration into the characteristics of the nursing work environment during the COVID-19 pandemic, along with initial evidence regarding the psychometric properties of scores on the C-NWE-PHE scale. The refined items of the scale were validated through expert consultation, rigorously tested using the SEM method, and further supported by score validity and reliability analysis. In comparison to the original 26-item C-NWE subscale designed for stable hospital settings, the C-NWE-PHE subscale identifies distinct characteristics that reflect the dynamic, challenging, uncertain, and unpredictable environments nurses encountered during the PHE. The identified characteristics include Workforce and Deployment Support, Leadership and Emergency Management, Autonomy and Empowerment, Teamwork and Collaboration, and Logistics and Humanistic Care. These concepts emerged from frontline experiences and were consistently observed among nurses throughout the testing process.

### 4.1. The Factor Structure of the C-NWE-PHE

The structure and characteristics identified in the C-NWE-PHE scale align with the multifaceted definition of the nursing work environment and conceptualize the situational factors specific to PHE contexts that is resource-limited and changeable rather than static and are closely associated with PHE preparedness and response [11, 12, 21, 27, 49]. Moreover, the study revealed robust relationships among the five elements of the nursing work environment, with association coefficients ranging from 0.85 to 0.97. This finding is consistent with previous research that explored the interconnectedness of these elements as challenges, opportunities, and managerial strategies for frontline environments, supporting nurses and promoting organizational performance [50, 51]. The significance of workforce support and effective leadership within the healthcare system is aligned with the achievement of resilience [52].

The Workforce and Deployment Support prioritizes both workforce capacity and allocation practices concerning deployed human resources and necessary work teams. It encompasses the work regulations and procedures for supporting nurses, making institutional support a crucial aspect of nurses' perception of a safe work environment. A key focus is on the willingness and suitability of allocating and deploying nurses to ensure optimal staffing and operational effectiveness, also as observed during the response to the Ebola outbreak [53]. This finding is highly relevant in the lessons gleaned from the COVID-19 pandemic, particularly regarding public health governance. It underscores the importance of coordinating service delivery, cultivating sustainable health workforces that leverage a diverse range of skills, establishing training pipelines, and implementing effective operating procedures at the organizational, regional, and national strategy levels [16, 18, 54]. Amid varied conditions encountered during the PHEs, witnessed across different countries, the utilization of nursing staff becomes crucial for coping with nursing shortages [6, 29, 37, 55]. This necessitates proactive measures and preparedness efforts, such as educationally prepared and deployment mechanisms, to address challenges that may arise due to administrative processes and the need for clear job descriptions and maintaining work-life balance [30, 56, 57].

The Leadership and Emergency Management subscale focuses on elements that nurses value in nurse leaders' efforts to foster a shared governance environment. This involves ensuring that leaders are accessible and facilitate collaborative decision-making, creating opportunities for nurses' voices, ideas, and suggestions to be involved and considered. During the pandemic, nursing leadership demonstrated high visibility and responsiveness towards the needs, concerns, suggestions, and contributions expressed by nurses [52, 58]. It is crucial to implement robust measures that unite and motivate nurses, ensuring that their voices are not only heard and respected but also acted upon [59, 60]. This approach to leadership is based on agile and transformative leadership styles, which are more effective in nursing culture compared to a command-and-control style [61]. Furthermore, nursing leadership that extends to higher levels within organizations, health systems, and governments can result in increased resource allocation to support frontline nurses in their work [62]. The significance of effective leadership at all levels of healthcare governance cannot be underestimated, as it plays a critical role in promoting a supportive and responsive work environment for nurses during challenging times like a PHE, as recognized across international experiences [11, 32, 55].

The Autonomy and Empowerment factor ensures that nurses are equipped with the necessary competence to deliver high-quality care to patients while also prioritizing their personal safety. This factor involves providing nurses with ample support for professional learning, recognizing the significant importance of training and reskilling programs, especially during emergency responses [63]. In contrast to the conventional approach of fostering a positive practice environment through ongoing professional development, the empowerment and facilitation of nurses' professional growth during times of crisis take a different approach, with an emphasis on appropriate education tailored for a “fit-for-purpose” workforce rather than lifelong learning [64]. This targeted focus on acquiring specific skills and knowledge required to handle critical situations equips nurses to confront challenges with confidence and leverage their expertise in acting and performing patient care effectively.

The factor of Teamwork and Collaboration plays a central role in the performance of healthcare organizations, involving not only healthcare professionals but also other members of the team. Effective teamwork and collaboration contribute to improved patient outcomes, streamlined workflow, and increased job satisfaction among nurses [65], particularly in demanding and time-sensitive emergency response situations [66] Nurses play a crucial role in the healthcare team, and their contributions are closely tied to the establishment of effective communication and efficient cooperation with other healthcare workers [67]. When nurses feel appreciated and their expertise is acknowledged, it fosters a positive and supportive working environment, which can ultimately lead to better patient care and organization performance. While these factors are commonly emphasized in describing the work environment in hospital settings, they become even more critical during emergency responses, where the traditional up and down hierarchy and status lines may be less emphasized [68]. By fostering a culture of teamwork and collaboration, healthcare organizations can create an environment that values the contributions of each team member and promotes open communication, mutual respect, and effective coordination [69, 70].

The factor of Logistics and Humanistic care within the organizational traits encompasses the provision of both physical and psychosocial support. Previous experiences and views of frontline healthcare workers during past pandemics have underscored the significance of adequate physical support, managing high workloads, handling long shifts, promoting adequate rest and recovery, and fostering positive relationships with the surroundings [71]. In this context, nurses expected additional benefits and spiritual rewards, beyond their regular salaries, as recognition for their dedicated contributions and extraordinary efforts made during these challenging times. Additionally, the demanding nature of frontline work has led nurses to desire adequate vacation time after fulfilling emergency tasks. Consistent with this, a review illustrated that organizations incorporated logistics support for nurses by ensuring the availability of personal protective equipment, adequate food and supplies, and also extending this support to the families of frontline workers to alleviate their concerns and provide a sense of security [72]. In addition to logistics support, emphasizing humanistic care for nurses has become a common practice to enhance their psychological well-being [73]. Nurse leaders who offer emotional support and empathy can significantly impact the overall morale and resilience of the nursing workforce [60, 74]. Empirical evidence from cross-sectional surveys indicates that nurses who perceive higher levels of organizational and social support, and demonstrate resilience, are more likely to report lower levels of anxiety related [75].

### 4.2. The Validity and Reliability of the C-NEW-PHE

The scale adaptation of the C-NWE was rigorously conducted following Heggestad's practical guideline and within the specific situational context of PHEs. An initial set of items was created based on the experiences of nurse managers in establishing a healthy work environment during the COVID-19 pandemic, taking into account the essential dimensions derived from previous descriptions of work environments in normal hospital settings, specifically considering the Chinese context [9, 22, 42, 43]. The scale items and construct were identified through collaboration with a research team and several nine-researcher workshops. Moreover, a Delphi expert panel method was employed to ensure content validity, resulting in acceptable I-CVI scores ranging from 0.85 to 1.00 and satisfactory S-CVI/Ave of 0.93, which affirms a strong agreement among experts regarding the relevance of the items and the scale's adequate content validity [76]. To further validate these items, the preliminary survey was conducted with nurses, confirming the suitability of the revised items.

During the development process, the internal consistency reliabilities and factor loadings of the assumed dimensions demonstrated satisfactory results. The scale validation of the C-NWE-PHE was performed by CFA using the SEM method, showing how relationships amongst items in the measure and the dimensions are consistent with the conceptual framework depicting the organizational traits of the work environment. The C-NWE-PHE exhibited a distinct factorial structure compared to both the original C-NWE scale and other existing instruments with varying content [20]. To further validate the scale's structure and model fits, future research should involve testing the C-NWE-PHE in various PHE events and across international healthcare organizations.

### 4.3. Limitations and Strengths

The generalizability of this study may be limited due to the sampling population, as it represents the first attempt to quantitatively assess workplace characteristics related to PHEs. Furthermore, the data collected from nurses relied on self-report measures, which can introduce response bias or social desirability bias. However, to address this limitation, we included data from nurse managers, validated the measures with experts, and conducted tests using the nurses' data. Additionally, as the primary aim was scale validation, a cross-sectional design was employed, which does not allow for establishing causal relationships between the work environment, nurses' characteristics, and nurses' outcomes. Further research is needed to explore their correlations with the performance of nursing management. It is worth noting that this study possesses several strengths, such as a rigorous scale adaptation process, a large sample size, multicenter data collection, comprehensive analysis, and a significant contribution to the field of nursing work environment during public health emergencies. The findings provide valuable evidence for understanding and assessing the work environment in such contexts, serving as a foundation for future research and improvement strategies in nursing practice.

### 4.4. Implications for Nursing Management

The theoretical constructs of the C-NWE-PHE scale provide valuable insights into nurses' perceptions of the nursing work environment during the PHEs, demonstrating robust validity and reliability. It can be potentially utilized in cross-sectional studies, prospective assessments of organizational traits, and interventional research. Future studies should not only concentrate on addressing acute burnout and reducing turnover intentions among nurses but should also prioritize the development and implementation of strategies that foster a healthy working environment conducive to nurses' well-being. To advance our understanding of the different constructs within the work environment and effectively implement improvements, concrete efforts and clear directions are needed for global applicability, including comparative studies across various workplaces to identify successful managerial measures. Further research aiming to pinpoint specific areas of improvement and interventions that target key factors such as workload management, professional development, leadership support, and collaboration can be designed. By considering the dynamic nature of the nursing work environment in PHE settings and understanding its interrelated components, healthcare organizations can better prepare for and respond to emergencies effectively.

## 5. Conclusions

This study employed a rigorous scale adaptation process that incorporated qualitative data, expert collaboration, and statistical analysis. Through this comprehensive approach, the reliability and validity of the C-NWE-PHE scale for assessing the work environment in the context of PHEs were ensured. Recognizing that the nursing workforce is an indispensable part of the health system and contributes to the resilience of organizations, it is imperative to invest in research and initiatives that foster a positive and supportive work environment for nurses.

## Figures and Tables

**Table 1:** Characteristics of the participants (*n* = 1059).

Characteristics	Participants (*n*)	(%)
Years of experience (mean, SD)	(9.2, 7.7)	
Sex		
Female	919	86.8
Male	140	13.2
Title level		
Primary	877	82.8
Intermediate	169	16.0
Deputy senior or above	13	1.2
Workload		
0–4 hours per day	382	36.1
5–8 hours per day	576	54.4
9–12 hours per day	97	9.2
13 hours and above per day	4	0.4
Workdays		
≤5 days per week	796	75.2
>5 days per week	263	24.8
Workplace (ever and current)		
Local residence community	112	10.6
Fangcang shelter	315	29.7
Public health centre	61	5.8
Designated hospital	345	32.6
Customs airport	20	1.9
Hainan province	176	16.6
Wuhan city	27	2.5
Other places	200	18.9

**Table 2:** Correlations among C-NWE-PHE subscales (*n* = 1059).

Subscales	*F*1	*F*2	*F*3	*F*4	*F*5	Alpha
*F*1 workforce and deployment support	1					0.97
*F*2 leadership and emergency management	0.94	1				0.97
*F*3 autonomy and empowerment	0.93	0.96	1			0.97
*F*4 teamwork and collaboration	0.93	0.92	0.97	1		0.98
*F*5 logistics and humanistic care	0.85	0.87	0.87	0.88	1	0.96
Overall scale	—	—	—	—	—	0.99

## Data Availability

The original contributions presented in the study are included in the article, and further inquiries can be directed to the corresponding author/s.
